# Prognostic Factors for Prolonged In-Hospital Stay in Patients with Heart Failure

**DOI:** 10.3390/medicina59050930

**Published:** 2023-05-12

**Authors:** Eglė Ignatavičiūtė, Diana Žaliaduonytė, Vytautas Zabiela

**Affiliations:** 1 Medical Faculty, Lithuanian University of Health Sciences, 44307 Kaunas, Lithuania; 2 Cardiology Department, Lithuanian University of Health Sciences, 44307 Kaunas, Lithuania; 3 Cardiology Department, Hospital of Lithuanian University of Health Sciences, 45130 Kaunas, Lithuania; 4 Kaunas Region Society of Cardiology, 50009 Kaunas, Lithuania; 5 Institute of Cardiology, Lithuanian University of Health Sciences, 44307 Kaunas, Lithuania

**Keywords:** heart failure, length of stay, hospitalization

## Abstract

*Background and Objectives*: Heart failure (HF) is a threatening health condition that is associated with an increasing prevalence and high expenses because of frequent patient hospitalizations. The purpose of this study was to evaluate the factors that influence the length of in-hospital stay in HF patients. *Materials and Methods*: A total of 220 patients (43.2% men), admitted to the Department of Cardiology, Kaunas Hospital of Lithuanian University of Health Sciences from the 1st of January 2021 to the 31st of May 2021, were included in this study. According to the length of in-hospital stay, patients were stratified into two groups: the first group’s length of stay (LOS) was from 1 to 8 days, and the second group’s LOS was 9 days or more. *Results*: The median LOS was 8 (6–10) days. Multivariate logistic regression analysis revealed five predictors as independent factors associated with prolonged hospitalization. These predictors included treatment interruption (OR 3.694; 95% CI 1.080–12.630, *p* = 0.037), higher value of NT-proBNP (OR 3.352; 95% CI 1.468–7.659, *p* = 0.004), estimated glomerular filtration rate (eGFR) ≤ 50 mL/min/1.73 m^2^ (OR 2.423; 95% CI 1.090–5.383, *p* = 0.030), systolic blood pressure (BP) ≤ 135 mmHg (OR 3.100; 95% CI 1.421–6.761, *p* = 0.004) and severe tricuspid valve regurgitation (OR 2.473; 95% CI 1.086–5.632, *p* = 0.031). *Conclusions*: Several variables were identified as significant clinical predictors for prolonged length of in-hospital stay in HF patients where treatment interruption, higher NT-proBNP value and lower systolic BP at admission were the most important.

## 1. Introduction

Heart failure (HF) is a threatening health condition that affects more than 64 million people in the world. As the population continues to age and the number of elderly people increases, HF prevalence is getting higher every year [1,2]. The estimated risk of developing congestive HF during the remaining course of life at the age of 40 years is 21.0% for men and 20.3% for women [3]. Ischemic heart disease (IHD) is a dominant underlying cause of HF (up to 42.3% of cases) [4]. However, the prevalence of IHD in HF patients is much higher—it takes up to 64% of all the cases and occurs more often in HF with reduced ejection fraction (HFrEF) and in HF with midrange ejection fraction (HFmrEF) than in HF with preserved ejection fraction (HFpEF) [2,5].

This health problem is associated with high expenditures because of frequent hospitalizations of the patients, as well as high morbidity and mortality of patients [6]. The estimated medical expenses for HF maintenance in the USA were more than 24 thousand US dollars per patient in a one-year period [7]. In addition to that, the evaluated price of HF patients’ lifetime care was 126819 US dollars per patient [8]. With an increasing number of HF cases, these estimations disclose a considerable economic problem in the healthcare system. Establishing prognostic factors for the prolonged length of stay (LOS) of hospitalized HF patients would be of value when dealing with the economic issues of HF.

Some characteristics predicting prolonged LOS in HF patients were revealed in earlier studies. The PROTECT study discovered that medical history of angina pectoris (AP) or diabetes mellitus, higher body mass index, heart rate higher than 90 beats per minute at admission, higher jugular venous pressure, higher blood urea nitrogen (BUN) and uric acid values were associated with longer LOS in acute HF patients [9]. The ESCAPE trial data revealed that older age, higher BUN, greater inferior vena cava diameter and lower sodium were independent predictors for longer than average LOS in patients with acute systolic HF [10]. Moreover, a cohort of more than 70 thousand HF patients showed that patients with prolonged LOS had more co-existing disorders [11].

The purpose of this study was to evaluate the factors that influence the length of in-hospital stay of patients who have HF.

## 2. Materials and Methods

### 2.1. Patients and Data Collection

The data of 220 patients (95 men and 125 women), admitted to the Department of Cardiology, Kaunas Hospital of Lithuanian University of Health Sciences from 1st of January 2021 to 31st of May 2021, were involved in this retrospective study. The inclusion criteria were patients with a diagnosis of HF. HF was defined as “a clinical syndrome with symptoms and or signs caused by a structural and/or functional cardiac abnormality and corroborated by elevated natriuretic peptide levels and or objective evidence of pulmonary or systemic congestion” [12]. Moderate or severe chronic kidney disease (CKD) was defined based on patients’ medical history or glomerular filtration rate (GFR) level below 60 mL/min/1.73 m^2^. Diabetes mellitus was defined based on patients’ medical history of present diabetes or the use of hypoglycemic medications. Thyroid dysfunction was defined based on patients’ medical history of hypothyroidism or hyperthyroidism. Iron deficiency anaemia (IDA) was defined based on a patient’s medical history or ferritin levels in the blood below normal. Data on other comorbidities were included in this study using the International Classification of Diseases (ICD) codes, which were known from the patients’ medical history.

We did not include patients who died within the first 3 days of in-hospital stay. Also, we did not include patients with iatrogenic complications, drug abuse or hospital infection or other visible reasons which could prolong in-hospital stay. 7.9% of eligible patients were excluded based on these criteria. The research was approved by the Bioethics Center of the Lithuanian University of Health Sciences (LUHS).

Study data were collected from medical records, including demographic characteristics, length of hospital stay, personal health history, clinical manifestations of HF, laboratory and echocardiographic findings. Patients were stratified into two groups regarding the median of patients’ LOS: the first group’s LOS was from 1 to 8 days (*n* = 115), and the second group’s LOS was 9 days or more (*n* = 105).

### 2.2. Statistical Analysis

Statistical analysis was performed using Statistical Package for the Social Sciences version 22.0 software. The normality of data was assessed with Kolmogorov–Smirnov test. If the continuous data met the normality criteria, Student’s *t*-test was used for the comparison of group means. Otherwise, Mann–Whitney U test was used for the comparison of groups. The continuous data following normal distribution were expressed as mean ± standard deviation (SD), while data following non-normal distribution were expressed as median (25th and 75th percentiles). Categorical variables were analyzed using the Pearson Chi-square test. Categorical data were expressed as frequency and percentage. The multivariate logistic regression analysis was performed to identify clinical predictors for prolonged LOS. The odds ratio (OR) and confidence intervals (CI) were calculated. Data differences were considered statistically significant at *p* < 0.05.

## 3. Results

### 3.1. Patients’ Demographic Characteristics and Medical History

The median length of in-hospital stays was 8 (6–10) days. A total of 115 (52.3%) patients were hospitalized for 1–8 days (group 1) and 105 (47.7%) patients were hospitalized for 9 or more days (group 2). HFrEF was known in 38 (33.6%) patients with shorter LOS and in 55 (52.4%) patients with longer LOS, HFmrEF—in 29 (25.7%) and 23 (21.9%) patients, and HFpEF—in 46 (40.7%) and 27 (25.7%) patients. HFrEF was statistically significant more frequently found in the prolonged LOS group, while HFpEF was more frequent in the shorter LOS group (*p* = 0.015). The demographic characteristics and medical history of comorbidities in different LOS groups were presented in Table 1.

There was no statistically significant difference regarding age, gender, BMI and etiology of IHD (previous myocardial infarction (MI), current stable or unstable AP) between the groups in terms of LOS. However, New York Heart Association (NYHA) class IV on admission was more frequent in patients with longer in-hospital stay, and NYHA class II on admission was more common in the shorter in-hospital stay group. Most of the pre-existing comorbidities (dyslipidemia, hypertension, atrial fibrillation (AF), aortic valve stenosis, thyroid dysfunction, moderate or severe CKD, chronic obstructive pulmonary disease (COPD), asthma, and gout) were similar in the groups. Meanwhile, pre-existing type 2 diabetes mellitus, IDA and previous strokes were more common in the 2nd LOS group (*p* = 0.049, *p* = 0.042 and *p* = 0.032, respectively).

Angiotensin II receptor blockers were more frequently prescribed in first group if compared to second (28 (24.3) vs. 14 (13.3) patients, *p* = 0.038), while intravenous and oral diuretics were statistically significant more often given to second group patients (86 (81.9) vs. 81 (70.4) patients, *p* = 0.047, and 87 (82.9) vs. 82 (71.3) patients, *p* = 0.043, respectively). Other drug prescriptions did not differ between different LOS groups.

A total of 15 patients died during the hospitalization: seven (6.1%) patients with shorter LOS and eight (7.6%) patients with longer LOS (*p* = 0.652).

### 3.2. Clinical Manifestations and Precipitating Factors of Heart Failure Decompensation

Clinical characteristics of HF on admission in different groups were shown in Table 2. Peripheral edema and crackles during auscultation were more frequently observed in patients with in-hospital stays of more than 8 days (*p* = 0.045 and *p* = 0.017, respectively).

Systolic and diastolic blood pressure (BP) were statistically significantly lower in the second group (t(3.8) = 0.992, *p* < 0.001 and t(2.3) = 0.989, *p* = 0.023, respectively), while heart rate was higher (*p* = 0.006). No significant differences were noticed between the groups when evaluating other parameters of the respiratory system.

Some precipitating factors for HF decompensation, such as infection or recent months and dose of diuretics used before hospitalization, did not differ between the groups, while treatment interruption was more frequently observed in the prolonged LOS group (*p* = 0.001).

### 3.3. Laboratory Findings

The distribution of laboratory findings in different groups was represented in Table 3. N-terminal pro-brain natriuretic peptide (NT-proBNP) was statistically significantly higher in HF patients who were hospitalized for more than 8 days (*p* < 0.001). Troponin T and creatinine levels were also elevated more in the prolonged LOS group (58.5 (26.0–134.4) ng/L vs. 33.3 (15.6–50.6) ng/L, *p* < 0.001 and 109.9 (85.6–151.0) µmol/L vs. 91.1 (78.1–115.3) µmol/L, *p* < 0.001, respectively). Hemoglobin, potassium, aspartate aminotransferase, alanine aminotransaminase, total cholesterol, low-density lipoprotein and glucose findings did not differ between the groups. The uric acid value was statistically significantly higher in the greater LOS group (644.9 ± 224.9 µmol/L vs. 505.0 ± 82.9 µmol/L, t(29) = −2.611, *p* = 0.014). Total protein level was similar among the groups, while serum albumin was more reduced in second-group patients (35.0 ± 5.0 g/L vs. 38.8 ± 5.1 g/L, t(51) = 2.534, *p* = 0.014).

### 3.4. Echocardiographic Findings

Echocardiographic findings in different groups were represented in Table 4. Left ventricular ejection fraction (LVEF) was statistically significantly lower in the second group, if compared to the first group (40 (25–50)% vs. 48 (40–50)%, *p* < 0.001). HFrEF was more often present in patients who had prolonged in-hospital stay (55 (52.4%) patients), if compared to shorter stay (38 (33.0%) patients) (*p* = 0.006).

Left ventricular posterior wall (LVPW) values were higher in the second group (11.4 (11.0–12.5) mm vs. 11.0 (10.0–12.0) mm, *p* = 0.006). Nevertheless, other echocardiographic findings of the left ventricle were similar in both groups. Left atrial volume index (LAVI) was higher in second-group patients (48.9 (40.1–59.2) mL/m^2^ vs. 43.4 (33.0–55.5) mL/m^2^, *p* = 0.006).

Mean pulmonary arterial pressure (mPAP) was higher in longer LOS group’s patients (37.5 (32.5–43.4) mmHg vs. 34.8 (27.0–40.6) mmHg, *p* = 0.026), while tricuspid annular plane systolic excursion (TAPSE) was lower in these patients (15.4 (13.5–19.8) mm vs. 17.8 (14.0–21.0) mm, *p* = 0.016).

Duration of in-patient treatment was statistically significantly associated with mitral valve (MV) regurgitation grade (*p* = 0.030). Mild MV regurgitation (grade I or I-II) was more frequent in patients who had a shorter LOS, if compared to longer LOS patients (30 (26.1%) patients vs. 15 (14.3%) patients), while moderate (grade II or II-III) had a higher incidence in prolonged LOS group (65 (61.9%) patients) when compared to shorter LOS (50 (43.5%) patients). LOS was also related to tricuspid valve (TV) regurgitation grade (*p* = 0.001). Mild TV regurgitation (grade I or I-II) was more frequent in shorter LOS group (30 (26.1%) patients vs. 10 (9.5%) patients), while severe TV regurgitation (grade III, III-IV or IV) was more common in longer LOS group (40 (38.1%) patients vs. 24 (20.9%) patients). The aortic valve regurgitation rate was similar in both groups. Other echocardiographic findings also did not differ in groups.

### 3.5. Prediction of Longer In-Hospital Stay

Multivariate logistic regression was performed with the purpose to investigate predictors for prolonged in-hospital stays. From the multivariate analysis, five predictors were identified as independent factors associated with prolonged hospitalization (Table 5). These predictors included treatment interruption, NT-proBNP > 4900 ng/L, eGFR ≤ 50 mL/min/1.73 m^2^ and systolic BP ≤ 135 mmHg at admission, severe TV regurgitation during 1st–2nd day of in-hospital treatment.

## 4. Discussion

Recognition of the patients, who are more likely to have a prolonged in-hospital stay, is significant for organizing effective care and appropriate distribution of resources in hospitals.

The findings of this research revealed that several variables were more prevalent in the longer LOS group. These characteristics include medical history of type 2 diabetes mellitus, IDA or previous stroke, as well as a determination of NYHA class IV at admission. Additionally, some clinical signs on the admission day (present peripheral edema, crackles during lungs auscultation, lower systolic or diastolic BP, tachycardia), laboratory changes (higher levels of NT-proBNP or creatinine and lower levels of albumin in blood) and echocardiographic properties (lower LVEF or TAPSE values, higher LVPW, mPAP or LAVI values, moderate MV and severe TV regurgitation) are also more likely to be found in patients with LOS greater than 8 days.

We used multivariate logistic regression to determine independent predictors for prolonged in-hospital stays. Five clinical characteristics were found, including treatment interruption, higher NT-proBNP (>4900 ng/L), lower eGFR (≤50 mL/min/1.73 m^2^), lower systolic BP (≤135 mmHg) at admission, and severe TV regurgitation found during echocardiographic examination. It is significant that most of these predictors may be evaluated in an emergency room by a primary physician. After evaluating the patient’s medical history, vital signs and laboratory tests, the physician could predict the prognosis of a patient and make more effective decisions (based on his risk for prolonged hospitalization). Some other studies found similar prognostic markers of prolonged LOS, while some variables were recognized as new risk factors for LOS in our research.

One study confirmed stroke as a risk factor associated with a higher chance of an abnormally prolonged hospital stay in HF patients (OR 1.27; 95% CI 1.23–1.31, *p* < 0.0001) [13]. Moreover, there is data that stroke in these patients is related to worse outcomes and elevated mortality rates [14]. In our study, previous stroke was not a significant predictor (*p* = 0.310).

Not much scientific data is available that lower systolic BP increases in-hospital stay. One study confirmed that relatively low systolic BP (<155 mmHg) on admission influenced the prolonged LOS in elderly patients with decompensated HF [15]. Systolic BP lower than 120 mmHg has been previously described as a forecaster of worse outcomes, as well as a higher chance of readmission and all-cause mortality in hospitalized HF patients [16,17]. Therefore, the antihypertensive treatment should be precisely considered and regulated in patients with established HF [18].

The presence of renal impairment was also confirmed as a predictor for prolonged LOS in several previous studies [19,20,21]. The presence of renal insufficiency (GFG < 60 mL/min) at admission (OD 1.25; 95% CI 1.03–1.52, *p* = 0.022) was a factor for prolonged in-hospital stay [20], as well as the development of renal impairment after treatment in hospital (OR 9.8; 95% CI 2.5–38.6, *p* = 0.001) [19]. Poor renal function is a prevalent problem in decompensated HF patients (especially in older patients), which is also associated not only with longer hospitalization but also with higher demands for diuretics [22].

NT-proBNP is a well-known predictor of HF, and higher values of NT-proBNP have associations with a higher incidence of all-cause in older patients with HF [23]. Higher values of NT-proBNP were presented as predictors for a prolonged in-hospital stay in other studies [21,24]

Increased heart rate can demonstrate increased activation of the intrarenal renin-angiotensin system (RAS) and sympathetic nervous system [23,24]. Activation of these systems is a pathophysiological mechanism of heart failure. The sympathetic nervous system is activated in mild congestive HF, while RAS—in severe and symptomatic congestive HF [25]. Moreover, studies show that tachycardia is a risk factor in patients with HF, as a higher heart rate increases the rate of cardiovascular death and admission to the hospital for worsening HF [26].

The remaining identified factors—treatment interruption and severe TV regurgitation—have not been documented before as predictors for longer LOS in HF patients. Non-compliance with pharmacological treatment was described as a frequent precipitating factor for HF (23% of cases) [25]. Tran et al. presented that discontinuation of guidelines-directed medical therapy was associated with lower rates of survival in hospitalized HF patients [26]. TV regurgitation is associated with elevated pulmonary artery pressures and increased overload in the major circulation circle. Severe TV regurgitation is an important factor for the increase of central venous pressure and systemic venous congestion, leading to the reduction of the renal blood flow, hepatic failure with increased hydrostatic pressure, and intra-abdominal edema with malabsorption [27]. To conclude, there is evidence that uncorrected TV regurgitation is associated with increased hospitalizations for HF and a higher rate of mortality in these patients [28].

However, some other predictors that extend in-hospital stay were found in the previous studies, such as older age [10], present IDA [29,30], type 2 diabetes mellitus [9,31], chronic pulmonary disease [20,32], a number of present comorbidities [11,33], higher BMI [9], presence of edema on admission [19], hypoxia (<90%) on admission [32], poor NYHA functional class [24,34], hypoalbuminemia [15,24,34], hyponatremia [10,20], higher levels of uric acid [9], higher BUN [9,10], greater inferior vena cava diameter [10].

Our research has potential limitations. The results of this study may underestimate the broader population of HF patients with ischemic etiology, because of potential selection bias, relatively small sample size and the single-center design. The conclusions of the results can be applicable to the number of HF patients with IHD.

The rate of hospitalizations in HF patients is assumed to increase up to 50% in the next 25 years [35]. It signals the importance of recognizing factors that predict prolonged LOS in these patients. Clinical findings that help to recognize patients who are likely to be discharged later, and can help to enhance the planning of hospital care and medical expenses.

## 5. Conclusions

Several variables were identified as significant clinical predictors for prolonged length of in-hospital stay in patients with HF where treatment interruption, higher NT-proBNP value and lower systolic BP at admission were the most important.

## Figures and Tables

**Table 1:** Demographic characteristics and medical history of patients with different length of stay.

Variables	≤8 Days	>8 Days	*p*-Value
Age, years, median (percentiles)	78 (69–86)	81 (73–85)	0.296
Gender:			1.000
- Male, *n* (%)	50 (43.5)	45 (42.9)	
- Female, *n* (%)	65 (56.5)	60 (57.1)	
BMI, kg/m^2^, mean ± SD	29.0 ± 7.2	29.4 ± 7.0	0.734
Previous MI, *n* (%)	30 (26.1)	26 (24.8)	0.944
Stable AP, *n* (%)	100 (87.7)	99 (94.3)	0.147
Unstable AP, *n* (%)	12 (10.4)	5 (4.8)	0.186
NYHA class on admission:			0.001
- Class II, *n* (%)	30 (26.1)	10 (9.5)	
- Class III, *n* (%)	82 (71.3)	83 (79.0)	
- Class IV, *n* (%)	3 (2.6)	12 (11.4)	
Comorbidities:			
- Dyslipidemia, *n* (%)	61 (53.0)	48 (45.7)	0.342
- Hypertension, *n* (%)	108 (93.9)	101 (96.2)	0.642
- AF, *n* (%)	74 (64.3)	80 (76.2)	0.077
- Aortic valve stenosis, *n* (%)	20 (17.4)	19 (18.1)	1.000
- Type 2 diabetes mellitus, *n* (%)	20 (17.4)	31 (29.5)	0.049
- Thyroid dysfunction, *n* (%)	16 (13.9)	17 (16.2)	0.777
- Moderate or severe CKD, *n* (%)	27 (23.5)	37 (35.2)	0.077
- COPD, *n* (%)	6 (5.2)	11 (10.5)	0.228
- Asthma, *n* (%)	6 (5.2)	8 (7.6)	0.651
- Iron deficiency anaemia, *n* (%)	10 (8.7)	20 (19.0)	0.042
- Gout, *n* (%)	19 (16.5)	14 (13.3)	0.637
- Previous stroke, *n* (%)	12 (10.4)	23 (21.9)	0.032
Prescribed treatment:			
- Beta blockers, *n* (%)	100 (87.0)	99 (94.3)	0.065
- ACEi, *n* (%)	74 (64.3)	76 (72.4)	0.201
- ARB, *n* (%)	28 (24.3)	14 (13.3)	0.038
- ARNi, *n* (%)	9 (7.8)	11 (10.5)	0.495
- MRA, *n* (%)	51 (44.3)	55 (52.4)	0.234
- Ivabradine, *n* (%)	2 (1.7)	2 (1.9)	0.927
- Intravenous diuretic, *n* (%)	81 (70.4)	86 (81.9)	0.047
- Oral diuretic, *n* (%)	82 (71.3)	87 (82.9)	0.043
- Statins, *n* (%)	50 (43.5)	41 (39.0)	0.505
- Antiaggregant, *n* (%)	35 (30.4)	25 (23.8)	0.270
- Anticoagulant, *n* (%)	92 (80.0)	92 (87.6)	0.127

BMI—body mass index; MI—myocardial infarction; AP—angina pectoris; NYHA—New York Heart Association; AF—atrial fibrillation; CKD—chronic kidney disease; COPD—chronic obstructive pulmonary disease; ACEi—angiotensin-converting enzyme inhibitor; ARBs—angiotensin II receptor blocker; ARNi—angiotensin receptor-neprilysin inhibitor; MRA—mineralocorticoid receptor antagonist.

**Table 2:** Main parameters at admission in different length of stay groups.

Variables	≤8 Days	>8 Days	*p*-Value
Peripheral edema, *n* (%)	71 (61.7)	79 (75.2)	0.045
Crackles, *n* (%)	73 (63.5)	83 (79.0)	0.017
Systolic BP, mmHg, mean ± SD	149 ± 25	136 ± 24	<0.001
Diastolic BP, mmHg, mean ± SD	86 ± 15	82 ± 16	0.023
Heart rate, b/min, median (percentiles)	80 (67–97)	86 (75–106)	0.006
RR, br/min, median (percentiles)	16 (16–17)	16 (16–18)	0.110
SpO_2_, %, median (percentiles)	96 (93–98)	95 (91–97)	0.168
Dose of diuretics before hospitalization:			0.027
Low dose of torasemide (<50 mg), *n* (%)	55 (47.8)	47 (44.8)	
High dose of torasemide (≥50 mg), *n* (%)	5 (4.3)	14 (13.3)	
Treatment interruption, *n* (%)	8 (7.0)	20 (19.0)	0.007
Any infection in recent months, *n* (%)	10 (8.7)	13 (12.4)	0.502

b/min—beats per minute; BP—blood pressure; RR—respiratory rate; br/min—breaths per minute, SpO_2_—oxygen saturation.

**Table 3:** Laboratory findings in different length of stay groups.

Variables	≤8 Days	>8 Days	*p*-Value
NT-proBNP, ng/L, median (percentiles)	2959 (1237–6476)	7765 (3352–13,891)	<0.001
TnT, ng/L, median (percentiles)	33.3 (15.6–50.6)	58.5 (26.0–134.4)	<0.001
Hemoglobin, g/L, mean ± SD	127.6 ± 20.8	122.0 ± 23.6	0.063
Potassium, mmol/L, median (percentiles)	4.35 (3.97–4.73)	4.34 (3.95–4.91)	0.929
Creatinine, µmol/L, median (percentiles)	91.1 (78.1–115.3)	109.9 (85.6–151.0)	<0.001
eGFR, ml/min/1.73 m^2^, median (percentiles)	66 (52–89)	53 (38–73)	0.001
AST, U/I, median (percentiles)	22.6 (19.2–36.6)	26.6 (20.2–45.1)	0.201
ALT, U/I, median (percentiles)	27.3 (13.9–43.4)	19.3 (13.6–43.2)	0.707
Uric acid, µmol/L, mean ± SD	505.0 ± 82.9	644.9 ± 224.9	0.014
Total cholesterol, mmol/L, mean ± SD	4.4 ± 1.2	4.0 ± 1.2	0.194
LDL, mmol/L, mean ± SD	2.7 ± 1.0	2.6 ± 1.0	0.487
Total protein, g/L, mean ± SD	60.5 ± 8.4	58.0 ± 6.0	0.208
Albumin, g/L, mean ± SD	38.8 ± 5.1	35.0 ± 5.0	0.014
Glucose, mmol/L, median (percentiles)	6.4 (5.7–7.6)	6.8 (6.1–8.1)	0.143

NT-proBNP—N-terminal pro-brain natriuretic peptide; TnT—troponin T; eGFR—estimated glomerular filtration rate; AST—aspartate aminotransferase; ALT—alanine aminotransaminase; LDL—low-density lipoprotein.

**Table 4:** Echocardiographic findings between groups of different lengths of stay.

Variables	≤8 Days	>8 Days	*p*-Value
LVEF, %, median (percentiles)	48 (40–50)	40 (25–50)	<0.001
LVMI, g/m^2^, median (percentiles)	112.4 (92.1–132.7)	115.1 (97.9–139.9)	0.312
LVEDD, mm, mean ± SD	49.6 ± 7.3	48.9 ± 9.4	0.569
IVS, mm, median (percentiles)	11 (10–13)	12 (11–13)	0.194
LVPW, mm, median (percentiles)	11.0 (10.0–12.0)	11.4 (11.0–12.5)	0.006
AoV Vmax, m/s, median (percentiles)	1.5 (1.3–1.9)	1.4 (1.2–2.0)	0.659
TV Vmax, m/s, median (percentiles)	3.0 (2.7–3.5)	3.2 (2.8–3.5)	0.134
mPAP, mmHg, median (percentiles)	34.8 (27.0–40.6)	37.5 (32.5–43.4)	0.026
TAPSE, mm, median (percentiles)	17.8 (14.0–21.0)	15.4 (13.5–19.8)	0.016
RWT, %, median (percentiles)	0.46 (0.42–0.52)	0.48 (0.43–0.55)	0.120
LA size in parasternal long-axis, mm, mean ± SD	46.4 ± 7.5	48.2 ± 8.3	0.135
LAVI, mL/m^2^, median (percentiles)	43.4 (33.0–55.5)	48.9 (40.1–59.2)	0.006
WMSI, median (percentiles)	1.0 (1.0–1.5)	1.0 (1.0–1.7)	0.892
E/A ratio, median (percentiles)	1.06 (0.76–2.27)	0.76 (0.63–1.45)	0.052
E/e‘ ratio, median (percentiles)	12.7 (7.9–15.8)	11.0 (9.2–13.9)	0.748
Diastolic dysfunction, *n* (%):			
- Impaired relaxation	24 (20.9)	21 (20.0)	0.193
- Pseudonormal	17 (14.8)	14 (13.3)	
- Restrictive filling	4 (3.5)	0 (0.0)	
Aortic valve stenosis, *n* (%)	20 (17.4)	19 (18.1)	1.000
MV regurgitation grade, *n* (%):			0.030
- Mild	30 (26.1) *	15 (14.3) *	
- Moderate	50 (43.5) *	65 (61.9) *	
- Severe	26 (22.6)	24 (22.9)	
TV regurgitation grade, *n* (%):			0.001
- Mild	30 (26.1) *	10 (9.5) *	
- Moderate	57 (49.6)	51 (48.6)	
- Severe	24 (20.9) *	40 (38.1) *	
AoV regurgitation grade, *n* (%):			0.987
- Mild	71 (62.8)	67 (63.8)	
- Moderate	19 (16.8)	17 (16.2)	
- Severe	3 (2.7)	3 (2.9)	

* Statistically significant differences were found between these groups (using z-test). LVEF—left ventricular ejection fraction; LVMI—left ventricular mass index; LVEDD—left ventricular end-diastolic diameter; IVS—interventricular septal thickness; LVPW—left ventricular posterior wall; AoV Vmax—peak aortic valve velocity; TV Vmax—peak velocity of regurgitation; mPAP—mean pulmonary arterial pressure; TAPSE—tricuspid annular plane systolic excursion; RWT—relative wall thickness, LA—left atrial; LAVI—left atrial volume index; WMSI—wall motion score index; MV—mitral valve; TV—tricuspid valve; AoV—aortic valve.

**Table 5:** Predictors for longer length of stay in hospital (multivariate logistic regression).

Variables	OR	95% CI	*p*-Value
Treatment interruption, *n* = 20	3.694	1.080–12.630	0.037
Previous stroke, *n* = 23	1.792	0.581–5.533	0.310
NT-proBNP > 4900 ng/L at admission, *n* = 55	3.352	1.468–7.659	0.004
eGFR ≤ 50 mL/min/1.73 m^2^ at admission, *n* = 45	2.423	1.090–5.383	0.030
Systolic BP ≤ 135 mmHg at admission, *n* = 50	3.100	1.421–6.761	0.004
Heart rate > 75 bpm at admission, *n* = 74	1.344	0.570–3.171	0.500
LVEF ≤ 40% during 1st–2nd day, *n* = 55	1.342	0.585–3.076	0.487
Severe TV regurgitation during 1st–2nd day, *n* = 40	2.473	1.086–5.632	0.031

OR—odds ratio; CI—confidence interval; NT-proBNP—N-terminal pro-brain natriuretic peptide; eGFR—estimated glomerular filtration rate; BP—blood pressure; bpm—beat per minute, LVEF—left ventricle ejection fraction; TV—tricuspid valve.

## Data Availability

Data are available upon request from corresponding authors.
